# Plant odor and sex pheromone are integral elements of specific mate recognition in an insect herbivore

**DOI:** 10.1111/evo.13571

**Published:** 2018-08-27

**Authors:** Felipe Borrero‐Echeverry, Marie Bengtsson, Kiyoshi Nakamuta, Peter Witzgall

**Affiliations:** ^1^ Corporación Colombiana de Investigación Agropecuaria (Agrosavia) Mosquera 4227373 Colombia; ^2^ Chemical Ecology Unit, Department of Plant Protection Biology Swedish University of Agricultural Sciences Alnarp 230 53 Sweden; ^3^ Graduate School of Horticulture Chiba University Matsudo Chiba 271‐8510 Japan

**Keywords:** Ecological speciation, premating sexual communication, reproductive isolation, specific mate recognition

## Abstract

Specific mate recognition relies on the chemical senses in most animals, and especially in nocturnal insects. Two signal types mediate premating olfactory communication in terrestrial habitats: sex pheromones, which blend into an atmosphere of plant odorants. We show that host plant volatiles affect the perception of sex pheromone in males of the African cotton leafworm *Spodoptera littoralis* and that pheromone and plant volatiles are not perceived as independent messages. In clean air, *S*. *littoralis* males are attracted to single synthetic pheromone components or even the pheromone of a sibling species, oriental cotton leafworm *S*. *litura*. Presence of host plant volatiles, however, reduces the male response to deficient or heterospecific pheromone signals. That plant cues enhance discrimination of sex pheromone quality confirms the idea that specific mate recognition in noctuid moths has evolved in concert with adaptation to host plants. Shifts in either female host preference or sex pheromone biosynthesis give rise to new communication channels that have the potential to initiate or contribute to reproductive isolation.

Specific mate communication and recognition, which is shaped during adaptation to natural habitats, involves both sex signals and environmental or habitat sensory cues and is under both sexual and natural selection (Paterson 1978, 1980; Endler 1992; Blows 2002; Boughman 2002; Scordato et al. 2014; Rosenthal 2017). In nature, females of phytophagous insects release sex pheromone into an atmosphere that is filled with plant volatiles. The effect of plant volatiles on the male moth behavioral response to sex pheromone has long been investigated (Landolt and Phillips 1997; Reddy and Guerrero 2004). Perception of sex and plant volatiles typically involves discrete peripheral input channels, and two different types of insect olfactory receptors, pheromone, and general odorant receptors, respectively (Krieger et al. 2004; Sakurai et al. 2004; Zhang and Löfstedt 2015). Integration of pheromone and plant volatile stimuli occurs in olfactory sensory neurons on the antennae in some species (Rouyar et al. 2015; Lebreton et al. 2017) and otherwise in the antennal lobe, the primary olfactory center in the insect brain (Namiki et al. 2008; Trona et al. 2010, 2013; Chaffiol et al. 2012, 2014; Hatano et al. 2015; Ian et al. 2017).

Curiously, the behavioral consequences of blending plant volatiles with sex pheromones differ among species and the plant chemicals investigated: plant volatiles can both synergize and antagonize the male response to sex pheromone (Dickens et al. 1990; Light et al. 1993; Yang et al. 2004; Schmidt‐Büsser et al. 2009; Trona et al. 2013; Badeke et al. 2016). A tentative explanation for this is that plant volatiles serve diverse behavioral roles, they signal plants for adult feeding, for mating or egglaying, or plants that that are not suitable as adult or larval food. Different messages conveyed by plant volatiles would account for different behavioral responses when blended with sex pheromone. This is evidenced by a response modulation according to internal physiological state in males and females of African cotton leafworm, *Spodoptera littoralis* (Lepidoptera, Noctuidae): unmated female moths are attracted to floral odorants for adult feeding, and soon after mating to cotton leaf volatiles for egg‐laying (Saveer et al. 2012). Male moths respond to host leaf volatiles only prior to mating, since these probably signal rendez‐vous sites (Kromann et al. 2015). Cotton leafworm moths further discriminate between volatiles of preferred and nonpreferred larval food plants (Thöming et al. 2013; Proffit et al. 2015) and between volatiles from healthy and damaged cotton plants (Zakir et al. 2013a,b; Hatano et al. 2015).

Inadequate plant stimuli, signaling damaged plants or nonhost plants that are unsuitable for oviposition, antagonize the male response to pheromone (Hatano et al. 2015; Badeke et al. 2016; Wang et al. 2016). This poses the question whether perception of host plant volatiles interacts with inadequate, heterospecific pheromone stimuli. Geographic distributions of the African and Oriental cotton leafworms, *S. littoralis* and *S. litura*, overlap in the Middle East, where cotton is a main larval host plant (Pogue 2002; Kergoat et al. 2012). The sex pheromones of the sibling species *S. littoralis* and *S. litura* are different, yet they are similar in composition and share several pheromone components: in a no‐choice situation in the laboratory, as many *S. littoralis* males are attracted to conspecific females and to *S. litura* females (Saveer et al. 2014). Since matings are prevented by incompatible genital morphology, we asked whether presence of the host plant cotton has an effect on male attraction to heterospecific pheromone.

Experiments with synthetic plant volatiles and pheromones, as well as live plants and pheromone‐releasing females show that males of *S. littoralis* best respond to a mixture of conspecific, complete sex pheromone, and volatiles of the larval food plant cotton. Attraction to heterospecific pheromone of the sibling species *S. litura* was much reduced in the presence of cotton volatiles, which demonstrates that mate recognition in cotton leafworm is mediated by a combination of plant volatiles and sex pheromone. This finding contributes to our understanding of olfactory‐mediated premating communication and reproductive isolation in insect herbivores.

## Materials and Methods

Laboratory colonies of African cotton leafworm *Spodoptera littoralis* and Oriental cotton leafworm *S. litura* (Lepidoptera, Noctuidea) were established with insects collected near Alexandria, Egypt and Chiba, Japan, respectively. Insect colonies were maintained with ca. 50 ovipositing females per generation. Every year, the *S. littoralis* lab population was separated by sex and interbred with 25–50 field‐collected males and females from Alexandria, Egypt. Insects were raised on a semisynthetic agar‐based diet (modified from Hinks and Byers 1976) under a 16L:8D photoperiod, at 24°C and 50–60% RH. Males and females were separated as pupae into 30 × 30 × 30 cm Plexiglas cages. Three‐day‐old unmated male moths were used in all bioassays.

Cotton seedlings, *Gossypium hirsutum* (cv. Delprim DPL 491), were grown singly in pots at 25°C and 70% RH in a greenhouse, under daylight and an added, artificial light source (metal halide, 400 W). Cotton plants used in wind tunnel assays had 8–12 fully developed true leaves and were 7–9 weeks old. Damaged plants were obtained by letting four, 24‐hour starved, fifth‐instar larvae feed on the plant for 4 hours prior to experiments. Larvae were removed before wind tunnel experiments. These growth conditions are similar to those used for cotton headspace analysis and behavioral tests reported in Saveer et al. (2012) and Borrero‐Echeverry et al. (2015).

### CHEMICALS

Volatiles released from cotton, which elicit either an antennal or a behavioral response in *S. littoralis* (Borrero‐Echeverry *et al*. 2015) were tested as single compounds: β‐myrcene (97% chemical purity; CAS #123‐35‐3; Fluka), (R)‐(+)‐limonene (95% chemical purity; CAS # 5989‐27‐5; Aldrich), (*E*)‐β‐ocimene (91% chemical purity; CAS #13877‐91‐3; Fluka), 4,8‐dimethyl‐1,3(*E*),7‐nonatriene (DMNT) (95% chemical purity; CAS #019945‐61‐0; a gift from Wittko Francke, Hamburg), (*Z*)‐3‐hexenyl acetate (99% chemical purity; CAS #3681‐71‐8; Aldrich), (R)‐(‐)‐linalool (95% chemical purity; CAS #126‐90‐9; Firmenich), (S)‐(+)‐linalool (95% chemical purity; CAS #126‐90‐9; Firmenich), and nonanal (90% chemical purity; CAS #124‐19‐6; Fluka). Two further compounds identified from maize headspace, another *S. littoralis* host plant (Bengtsson et al. 2006; Thöming et al. 2013) were included in the experiments with single plant volatiles: α‐farnesene (>90% chemical purity; CAS #502‐61‐4; Bedoukian) and β‐farnesene (>90% chemical purity; CAS #18794‐84‐8; Bedoukian).

The *S. littoralis* pheromone components, (*Z*,*E*)‐9,11‐tetradecadienyl acetate (Z9,E11‐14Ac) (main pheromone compound), (*Z*)‐9‐tetradecenyl acetate (Z9‐14Ac), (*Z*,*E*)‐9,12‐tetradecadienyl acetate (Z9,E12‐14Ac), were purchased from Pherobank and (*E*,*E*)‐10,12‐tetradecadienyl acetate (E10,E12‐14Ac) was provided by David Hall (Greenwich, UK). Isomeric purity was >96.3% for the dienic compounds, and >99.1% for Z9‐14Ac. These four components were consistently found in pheromone gland extracts (Saveer et al. 2014; El‐Sayed 2017). The solvent used for diluting synthetic compounds was redistilled ethanol (100% pure, Labscan, Malmö, Sweden).

### WIND TUNNEL BIOASSAY

Wind tunnel experiments were performed in a Plexiglas wind tunnel (180 × 90 × 60 cm) following the protocol of Borrero‐Echeverry et al. (2015). Briefly, males and females were kept in separate rooms to avoid pre‐exposure to pheromone before experiments. One hour before experiments, moths were transferred individually to 2.5 × 12.5 cm glass tubes closed with gauze. Tests were carried out between 1 and 4 hours after the onset of scotophase. The wind tunnel was illuminated from above and the side (6 lux), moths were flown at a wind speed of 30 cm/s, at 24 ± 2°C air temperature and 60 ± 10% RH. Incoming and outgoing air was filtered with active charcoal. Moths for every treatment (*N* = 50) were released individually from glass tubes at the downwind end of the tunnel. Males were scored for anemotactic upwind flight (Carde and Baker 1984) over 150 cm, from the release tube to the odor source.

Synthetic odor blends were delivered from the center of the upwind end of the wind tunnel from a piezo‐electric sprayer (El‐Sayed et al. 1999; Becher et al. 2010). Samples were loaded into a 1‐mL glass syringe operated by a microinjection pump (CMA Microdialysis AB, Solna, Sweden) that delivered test solutions at a constant rate of 10 μL/min through Teflon tubing into a glass capillary with a narrow, elongated tip. The capillary was attached to a piezo‐ceramic disk, which produced an aerosol that was carried downwind. A glass cylinder (95 mm diameter × 100 mm height), covered by a fine metal mesh (pore size 2 mm) was placed in front of the capillary as landing platform. Live plants were placed at the upwind end of the wind tunnel. In experiments with calling, pheromone‐releasing females, three calling females were placed downwind from plants in glass tubes covered at both ends with a mesh.

We tested the main pheromone compound, Z9,E11‐14Ac and a 4‐component synthetic pheromone blend of Z9,E11‐14Ac, Z9‐14Ac, E10,E12‐14Ac, and Z9,E12‐14Ac, in a 100:30:20:4 proportion. The release rate of the main compound Z9,E11‐14Ac was 100 pg/min, corresponding to the amount of pheromone emitted by calling females (Saveer et al. 2014). Males were further tested with pheromone‐releasing *S. littoralis* and *S. litura* females. Single plant compounds were released at a rate of 10 ng/min, and a 4‐component plant volatile blend containing nonanal, (R)‐(+)‐limonene, (*Z*)‐3‐hexenyl acetate, (*E*)‐β‐ocimene in a 33:12:33:23 proportion (Borrero‐Echeverry et al. 2015) was also released at 10 ng/min. A 5‐component plant volatile blend, mimicking herbivore damage, was formulated by adding DMNT at 10 ng/min to the 4‐component blend (Hatano et al. 2015). Males were further tested with undamaged cotton plants and plants on which 5th‐instar larvae of *S. littoralis* had been feeding during 4 h. Males were flown to single sources of plant volatiles and pheromones, and to combinations of both.

### STATISTICAL ANALYSIS

Generalized linear models (GLM) with a binomial distribution were used to analyze the number males attracted to different stimuli. Upwind flight was used as the target effect. Post‐hoc Wald pairwise comparison tests were used to identify differences between treatments. All statistical analysis was carried out using R (R Core Team 2013), using the NLME package (Pinheiro et al. 2018).

## Results

When cotton volatiles were tested one by one, only α‐farnesene elicited significant upwind flight attraction in *S. littoralis* males (*z* = 2.07; *P* = 0.039) (Table 1). All of these plant volatiles significantly reduced male attraction, when added to the main pheromone compound Z9,E11‐14Ac. In stark contrast, seven of these plant volatiles did not affect male attraction when mixed with the complete, four‐component synthetic sex pheromone. Only three volatiles reduced attraction when mixed with the four‐component sex pheromone blend: DMNT was the strongest antagonist (*z* = 4.60; *P* < 0.001), followed by (*E*)‐β‐ocimene (*z* = 2.38; *P* = 0.017) and (*Z*)‐3 hexenyl acetate (*z* = 1.80; *P* = 0.072) (Table 1). Larval feeding on cotton leaves strongly increases release of DMNT, which has been shown to interfere with perception of the main pheromone compound Z9,E11‐14Ac (Hatano et al. 2015).

**Table 1:** Male cotton leafworm *S. littoralis* upwind flight attraction to synthetic cotton volatiles (Loughrin et al. 1995; Saveer et al. 2012; Yang et al. 2013) and sex pheromone compounds (Saveer et al. 2014; El‐Sayed 2017)

	Male upwind flight attraction [%]		
Pheromone added	None	Main pheromone compound^a^	4‐Component sex pheromone blend^b^
		48	64
Plant compounds^c^			
α‐Farnesene	14^*^	24^*^	76
Nonanal	0	18^*^	72
(R)‐(+)‐Limonene	6	18^*^	64
(S)‐(+)‐Linalool	8	14^*^	62
β‐Farnesene	2	20^*^	58
(R)‐(+)‐Linalool	0	0^*^	54
β‐Myrcene	8	14^*^	50
(*Z*)‐3‐Hexenylacetate	0	8^*^	46^*^
(*E*)‐β‐Ocimene	4	22^*^	40^*^
DMNT^d^	0	2^*^	16^*^

^a^Z9,E11‐14Ac, release rate 100 pg/min.

^b^100:30:20:4‐blend of Z9,E11‐14Ac, Z9‐14Ac, E10,E12‐14Ac and Z9,E12‐14Ac, release rate 100 pg/min.

^c^release rate 10 ng/min.

^d^4,8‐dimethyl‐1,3(*E*),7‐nonatriene.

Single cotton volatiles were tested alone, in mixtures with the *S. littoralis* main pheromone compound, and with an optimized, four‐component synthetic sex pheromone blend. Asterisks show significant differences between attraction to pheromone alone and pheromone blended with single cotton volatile compounds; α‐farnesene was the only cotton volatile to elicit significant attraction by itself (binomial GLM and *post‐hoc* Wald pairwise comparison; *n* = 50).

**Figure 1:**
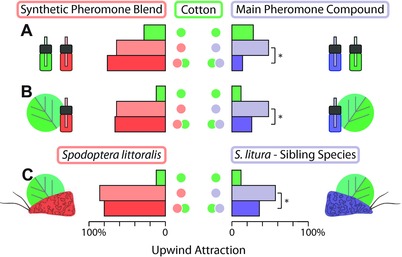
Male *S. littoralis* upwind flight attraction toward pheromone and cotton volatiles. The top two bars of each subplot show attraction to single plant and pheromone stimuli, respectively, while the third bar shows attraction to the combination of the respective plant and pheromone stimulus. The stimuli are main pheromone compound alone, an optimized four‐component *S. littoralis* synthetic sex pheromone blend (A, B) or pheromone‐reasing *S. littoralis* or *S. litura* females (C), in combination with a synthetic cotton volatile blend (A) or a live cotton plant (B, C). Bars with asterisks are significantly different from attraction to pheromone control (binomial GLM and *post‐hoc* Wald pairwise comparisons; *n* = 50).

This differential effect of complete versus incomplete pheromone on male attraction, when mixed with plant compounds, was confirmed by experiments using a 4‐component cotton volatile blend, instead of single cotton volatiles. Attraction to a combination of this cotton blend and the main pheromone compound was significantly reduced, compared with attraction to pheromone alone (*z* = 3.73; *P* < 0.001), while a combination of the same cotton blend with four‐component pheromone did not reduce attraction (Fig. 1A). An undamaged cotton plant produced the same result: male attraction was significantly reduced to the plant in combination with the main pheromone compound (*z* = 2.21; *P* = 0.027), and not with the complete pheromone blend (*z* = 0.21; *P* = 0.834; Fig. 1B).

We next replaced synthetic with authentic sex pheromone, released by live calling conspecific females or by females of the sibling species *S. litura*. Both species share main pheromone components, which explains *S. littoralis* male attraction to pheromone‐releasing *S. litura* females in clean air (Fig. 1C; Saveer et al. 2014). However, these two pheromone blends differ in composition and males are capable of discriminating conspecific from heterospecific pheromone, since they prefer conspecific over heterospecific *S. litura* females in choice tests (Saveer et al. 2014). Tests with pheromone‐releasing females on cotton plants confirm the results obtained with synthetic pheromone: adding cotton to *S. litura* females significantly reduced male upwind flights, compared to calling *S. litura* females alone (*z* = 1.99; *P* = 0.046) (Fig. 1C).

Lastly, we examined the effect of volatiles from cotton challenged by larval feeding on male sex pheromone attraction. We used a synthetic cotton blend and cotton plants on which *S. littoralis* larvae had been feeding. The synthetic blend mimicking damaged cotton and a cotton plant damaged by feeding larvae both significantly reduced attraction to the 4‐component synthetic pheromone, respectively (*z* = 2.21; *P* = 0.027 and *z* = 2.95; *P* = 0.003; *z* = 3.90). Damaged cotton plants even reduced attraction to calling *S. littoralis* females (*z* = 3.992; *P* < 0.001) (Table 2).

**Table 2:** Male *S. littoralis* upwind flight attraction towards blends of *S. littoralis* pheromone and volatiles of cotton plants damaged by larval feeding

	Male upwind flight attraction [%]		
Pheromone stimulus	Main pheromone compound^a^	4‐Component sex pheromone blend^b^	Pheromone‐releasing *S*. *littoralis* female
	48^*^	64^*^	88^*^
Plant stimulus added			
Damaged cotton volatiles c	2	34	—d
Damaged cotton plant	10	24	48

^a^Z9,E11‐14Ac, release rate 100 pg/min.

^b^100:30:20:4‐blend of Z9,E11‐14Ac, Z9‐14Ac, E10,E12‐14Ac and Z9,E12‐14Ac, release rate 100 pg/min.

^c^33:12:33:23‐blend of nonanal, (R)‐(+)‐limonene, (Z)‐3‐hexenyl acetate, (E)‐β‐ocimene (release rate 10 ng/min), and 4,8‐dimethyl‐1,3(*E*),7‐nonatriene (DMNT; release rate 10 ng/min).

^d^not tested.

Males were flown to the main pheromone compound, a four‐component sex pheromone blend or a pheromone‐releasing *S. littoralis* female, together with a synthetic blend of herbivore‐damaged cotton volatiles, or a live cotton plant damaged by *S. littoralis* larval feeding. Asterisks show significant differences between attraction to pheromone and to pheromone and plant stimulus (binomial GLM and *post‐hoc* Wald pairwise comparisons; *n* = 50).

## Discussion

That mate finding is elicited by an ensemble of sexual and environmental odorants, which mutually affect each other, provides a new perspective of premating communication in moths. The behavioral role of plant volatiles in male moth sexual behavior has not been entirely resolved. It has been proposed that host plant volatiles mediate male attraction to mating sites either by themselves, before the onset of pheromone release by females, or by synergizing the response to sex pheromone (Landolt and Phillips 1997; Reddy and Guerrero 2004; Beyaert and Hilker 2014). In some species, host plant volatiles increase male attraction toward sex pheromone (Dickens et al. 1993; Light et al. 1993; Yang et al. 2004; Schmidt‐Büsser et al. 2009; Varela et al. 2011; von Arx et al. 2012), whereas they produce an antagonistic effect in other species (Pregitzer et al. 2012; Jung et al. 2013; Party et al. 2013; Rouyar et al. 2015). It is conceivable that volatiles from nonhost plants (Wang et al. 2016), volatiles from damaged plants, such as DMNT (Fig. 1C; Hatano et al. 2015), or floral odorants, such as β‐ocimene, which signal adult food sources (Fig. 1C; Kroman et al. 2015; Zakir et al. 2017) do not synergize pheromone attraction. It seems counter‐intuitive, on the other hand, that volatiles of larval food plants would inhibit male attraction to conspecific female sex pheromone, since many moths mate on their respective host plants, where females oviposit.

Our results offer an explanation for this conundrum. Host plant volatiles reduce male attraction to heterospecific or incomplete synthetic pheromone (Table 1, Fig. 1). This compares to findings in grapevine moth *Lobesia botrana*, where host plant volatiles increased male attraction to an optimized pheromone blend, but decreased attraction to a single pheromone component (Sans et al. 2016). On the other hand, it has already been shown that volatiles from non‐host plants or less preferred host plants reduce attraction to conspecific pheromone in *S*. *littoralis* and fall armyworm *S*. *frugiperda* (Anderson et al. 2013; Binyameen et al. 2013; Thöming et al. 2013; Unbehend et al. 2013).

Cotton is a common plant host for the sibling species *S. littoralis* and *S. litura*, which are distributed in Africa and the West Palearctic and in Asia, respectively; their distributions overlap in the Middle East (Pogue 2002; Kergoat et al. 2012). Males of the African cotton leafworm *S. littoralis* are attracted to females of both species, but prefer conspecific females in choice tests; heterospecific matings with *S.litura* females are prevented by genital morphology (Saveer et al. 2014). Presence of the plant host accentuates differences between conspecific and heterospecific sex pheromones (Fig. 1B,C). This interaction between plant cues and sex signals is consequential, since it facilitates specific mate finding and recognition in closely related species, which frequently use pheromone blends that share compounds and partially overlap in composition (El‐Sayed 2017). Sex pheromones typically consist of a blend of several compounds that have been shown to function as a unit (Linn et al. 1986). Our findings demonstrate that this laboratory‐derived concept must be updated to accommodate the role of host plant volatiles in pheromone communication: it is the ensemble of social signals and environmental cues that mediates mate finding and recognition in natural environments.

Changes in female pheromone production and the corresponding shift in male preference are driven by sexual selection. Divergence of sex pheromone blends has been documented in populations of the same species (Cardé et al. 1978; Malausa et al. 2005; Groot et al. 2008; Velasquez‐Velez et al. 2011) or in sibling species (Lofstedt and van der Pers 1985; Bengtsson et al. 2014; Saveer et al. 2014). According to the “asymmetric tracking” hypothesis, males track changes of the female pheromone composition and rather quickly develop a preference for new pheromone blends (Phelan 1992; Heckel 2010; Droney et al. 2012). Such changes are believed to enable sympatric speciation in moths through premating behavioral isolation (Smadja and Butlin 2009; M'Gonigle et al. 2012).

Host plant shifts in females, on the other hand, are driven by natural selection. If shifts in female host plant preference lead to disruptive selection on host use, they may lead to speciation with little or no changes in pheromone composition (Lofstedt and van der Pers 1985; Witzgall et al. 1991; Drès and Mallet 2002; Bengtsson et al. 2006; Leppik and Frérot 2012). Matsubayashi et al. (2010) suggest that changes in host plant preference may lead to premating isolation based solely on a reduced probability of encounters between populations associated with different hosts. In polyphagous species where populations have generalized diets, individuals may have preferences for a particular host plant and may be subject to selective pressures that may lead to diversification (Bolnick et al. 2003; Rueffler et al. 2006). The recent finding that host plant choice in *S. littoralis* is modified by larval experience or adult learning (Proffit et al. 2015) supports a scenario where individual preference could lead to host plant shifts and initiate divergence.

If host plant volatiles affect pheromone perception, it follows that male moth pheromone detection and mate finding is under combined sexual and natural selection. Traits combining local adaptation and mating decisions have been termed “magic traits” since they facilitate phylogenetic divergence, especially in insect herbivores (Gavrilets 2004; Pfennig and Pfennig 2009; Smadja and Butlin 2009; Servedio et al. 2011; Safran et al. 2013; Thibert‐Plante and Gavrilets 2013; Rebar and Rodríguez 2015). Under sympatric conditions, natural selection alone is unlikely to lead to speciation due to random mating, however, if selection acts on both habitat and mate preference simultaneously, speciation is far more likely to occur.

Our system demonstrates a mechanism where the behavioral consequences of shifts in sex pheromone biosynthesis are reinforced by host plant volatiles. New pheromone communication channels may give rise to reproductive isolation, especially in populations diverging onto new host plants. Mate finding mediated by a combination of pheromones and host plant volatile signatures will reinforce premating barriers when a population undergoes disruptive selection (Ritchie 2007; Butlin et al. 2012; Boughman and Svanbäck 2016). This adds further support to the view that sympatric speciation has contributed to shaping the tremendous diversity of phytophagous insects (Tauber and Tauber 1989; Berlocher and Feder 2002; Drès and Mallet 2002; Forbes et al. 2017).

## DATA ACCESSIBILITY

Data available at the Dryad Digital Repository https://doi.org/10.5061/dryad.v54v8.

Associate Editor: G. Rosenthal

Handling Editor: P. Tiffin

## Supporting information

Treatment

Single host plant volatiles
Main pheromone component (MPC) + single host plant volatiles
4 component pheromone (4CP) + single host plant volatiles
Main pheromone component (MPC) + plant blends
4 component pheromone (4CP) + plant blends
*S. littoralis* females
*S. litura* females
