# Supplementary material for: Plant odor and sex pheromone are integral elements of specific mate recognition in an insect herbivore
Source: Evolution. 2018 Aug 27;72(10):2225–33. doi: 10.1111/evo.13571 (PMC6220987; doi:10.1111/evo.13571)
Supplement: Supplementary file 2 — Single host plant volatiles Main pheromone component (MPC) + single host plant volatiles 4 component pheromone (4CP) + single host plant volatiles Main pheromone component (MPC) + plant blends 4 component pheromone (4CP) + plant blends S. littoralis females S. litura females [file EVO-72-2225-s002.docx]

**Single host plant volatiles**

| Treatment | DMNT (6) | Ocimene (15) | ß-Myrcene  (17) | R-linalool  (21) | S-linalool  (23) | Z3-hexenly acetate  (25) |
| --- | --- | --- | --- | --- | --- | --- |
| Pure air  (0) | z=0.000  p=1.000 | z=0.575  p=0.565 | z=1.275  p=0.202 | z=0.000  p=1.000 | z=1.275  p=0.202 | z=0.000  p=1.000 |

continued

**Main pheromone component (MPC) + single host plant volatiles**

| Treatment | DMNT (6) | Ocimene (15) | ß-Myrcene  (17) | R-linalool  (21) | S-linalool  (23) | Z3-hexenly acetate  (25) |
| --- | --- | --- | --- | --- | --- | --- |
| Pure air  (0) | z=0.000  p=1.000 | z=0.575  p=0.565 | z=1.275  p=0.202 | z=0.000  p=1.000 | z=1.275  p=0.202 | z=0.000  p=1.000 |

| Treatment | MPC + DMNT (7) | MCP + Ocimene (16) | MCP + ß-Myrcene (18) | MCP + R-linalool (22) | MCP + S-linalool (24) | MCP + Z3-hexenly acetate  (26) |
| --- | --- | --- | --- | --- | --- | --- |
| MPC  (2) | z=-3.663  p=0.003 | z=-2.894  p=0.004 | z=-3.733  p=0.0001 | z=-3.663  p=0.0003 | z=-3.733  p=0.0002 | z=-4.158  p<0.00001 |

continued

| Treatment | MCP + R-limonene  (28) | MCP + Nonanal  (30) | MCP + a-Farnesene  (32) | MCP + b-farnesene  (34) |
| --- | --- | --- | --- | --- |
| MPC  (2) | z=-3.331  p<0.0001 | z=-3.538  p=0.0004 | z=-2.668  p=0.008 | z=-3.116  p=0.0018 |

**4 component pheromone (4CP) + single host plant volatiles**

| Treatment | 4CP + R-linalool (38) | 4CP + S-linalool (39) | 4CP + a-Farnesene  (40) | 4CP + b-Farnesene  (41) | 4CP + DMNT (42) | 4CP + Ocimene (43) |
| --- | --- | --- | --- | --- | --- | --- |
| 4CP  (36) | z=-1.409  p=0.1589 | z=0.000  p=1.000 | z=1.303  p=0.193 | z=-0,412  p=0.680 | z=-4.602  p<0.0001 | z=-2.278  p=0.0174 |

| Treatment | R-limonene  (27) | Nonanal  (29) | a-Farnesene  (31) | b-farnesene  (33) |
| --- | --- | --- | --- | --- |
| Pure air  (0) | z=1.275  p=0.202 | z=0.000  p=1.000 | z=2.066  p=0.039 | z=0.000  p=1.000 |

continued

| Treatment | 4CP + ß-Myrcene  (44) | 4CP + Z3-hexenly acetate  (45) | 4CP + R-limonene  (46) | 4CP + Nonanal  (47) |
| --- | --- | --- | --- | --- |
| 4CP  (36) | z=-1.409  p=0.1589 | z=-1.799  p=0.0721 | z=0.000  p=1.000 | z=0.856  p=0.392 |

**Main pheromone component (MPC) + plant blends**

| Treatment | 4C blend + MPC  (53) | 4C blend + DMNT + MPC  (55) | Cotton plant + MPC  (59) | Dam. Cotton Plant + MPC  (61) |
| --- | --- | --- | --- | --- |
| MPC  (2) | z=-3.733  p<0.0001 | z=-3.331  p<0.0001 | z=-2.208  p=0.027 | z=-4.055  p<0.0001 |

**4 component pheromone (4CP) + plant blends**

| Treatment | 4C blend + 4CP  (54) | 4C blend + DMNT + 4CP  (56) | Cotton plant + 4CP  (60) | Dam. Cotton Plant + 4CP  (62) |
| --- | --- | --- | --- | --- |
| 4CP  (36) | z=1.303  p=0.193 | z=-2.953  p=0.003 | z=0.210  p=0.834 | z=-3.899  p<0.0001 |

***S. littoralis* females**

| Treatment | S. littoralis + cotton plant  (65) | S. littoralis + Dam. cotton plant  (66) |
| --- | --- | --- |
| Calling *S. littoralis*  (63) | z=-0.835  p=0.404 | z=-3.992  p<0.0001 |

***S. litura* females**

| Treatment | S. litura + cotton plants |
| --- | --- |
| Calling *S. litura*  (64) | z=-1.992  p=0.046 |
